# Fit for Purpose? The Suitability of Oral Health Outcome Measures to Inform Policy

**DOI:** 10.1177/23800844231189997

**Published:** 2023-08-09

**Authors:** T.M. Nguyen, H. Rogers, G.D. Taylor, U. Tonmukayakul, C. Lin, M. Hall, H. Calache, C. Vernazza

**Affiliations:** 1 Deakin Health Economics, Institute for Health Transformation, Deakin University, Melbourne, VIC, Australia; 2 Public Health & Preventive Medicine, Faculty of Medicine, Nursing and Health Sciences, Monash University, Melbourne, VIC, Australia; 3 Dental Health Services Victoria, Carlton, VIC, Australia

**Keywords:** dental caries, population health, public health dentistry, health economics, patient preference, health-related quality of life

## Abstract

Measuring outcomes for oral health is frequently undertaken using the decayed, missing, and filled teeth (DMFT) index. DMFT measures dental caries experience. As a condition-specific outcome, it is not relatable to consumers, nor does it capture the impact of dental caries on quality of life or outcomes that matter most to consumers. Efficient resource allocation should include patient values. It is crucial to use measurement instruments that record consumer values or preferences for oral health and oral diseases. These measures are often called patient-reported outcome measures and/or patient-reported experience measures. Outcome measures relevant to patients are recommended to influence and inform policy decision-making for oral health.

**Knowledge Transfer Statement:**

Oral health research and program evaluation should consider alternative outcome measures for population oral health other than the DMFT index.

## Introduction

Measuring oral health outcomes in clinical practice and research is important for oral disease surveillance, evaluation of population and clinical interventions, and to inform oral health policy. Dental caries experience has predominantly been measured using the decayed, missing, and filled teeth (DMFT) index. This invited commentary discusses limitations of using the DMFT index in economic evaluations, explores alternative approaches to oral health measurement, and provides recommendations to inform policy decision-making.

## Limitations of the DMFT

The DMFT index is a numerical count of teeth affected by dental caries and includes decayed teeth and teeth extracted or restored due to dental caries. This condition-specific outcome measure is not directly relevant to consumers’ overall health and does not necessarily indicate the relative state of oral health or disease severity (Broadbent and Thomson 2005).

Dental caries incidence is typically quantified by the incremental mean value of DMFT in the population between age cohorts (GBD 2017 Oral Disorders Collaborators et al. 2020). However, consistency in its measurement is threatened by variations of the DMFT index (Rogers et al. 2019) (e.g., severity of caries/measurement of surfaces) and disregards the impact on consumers affected by the disease (Nguyen et al. 2022). In addition, mean DMFT values can often hide a skewed distribution, ignoring potential inequalities.

The DMFT index does not reflect the impact dental caries has on a person’s quality of life or the value and preferences consumers place on their oral health and oral health choices, in relation and addition to a broader context of health. As an essential input for resource allocation, it is important to measure this value and/or preference. Such preferences can be measured in terms of disability-adjusted life years (DALYs) or quality-adjusted life year (QALYs). DALYs and QALYs are commonly used in other health fields but not well applied in oral health.

## Alternative Approaches Measuring Oral Health

A methodology developed through the Global Burden of Disease (GBD) project enables an estimation of DALYs from DMFT (GBD 2017 Oral Disorders Collaborators et al. 2020). DALYs is the number of years free from disability and premature death related to a specific health condition, where 0 represents full health and 1 represents death. DALYs permit ranking across many disease and health conditions, within and between countries.

Although DALYs have some advantages, they do not consider the nuances of consumer preferences (i.e., self-reported/proxy and child/adult perspectives). This is because DALYs use preferences based on expert opinion, to quantify the value of health loss. Another approach is measuring a preference-based quality-of-life (QoL) measure, which gives an inversed index range between 0 (death) and 1 (full health) for being in a health state. QoL instruments can be used to calculate QALYs, a preferred measure by many health policy decision-making bodies, internationally.

Previous reviews have explored whether existing QoL instruments can be used with children (Hettiarachchi et al. 2019) and adults (Riva et al. 2022) for oral health. However, most generic QoL instruments currently used in oral health research do not measure preference-based QoL and instead have simple summative scoring systems, which preclude the generation of QALYs.

A preference-based measure is composed of 2 parts: a descriptive system that comprises a series of characteristics relevant for the condition and/or population and a value set, or scoring tariff, for all the health states encompassed by the descriptive system, based on the preferences of the population (Goodwin and Green 2016). When the questions within a preference-based measure are completed by consumers, the scoring algorithm converts responses into a single value, or index, which can be used to calculate QALYs.

Concerns have been raised regarding the relevance and sensitivity of commonly used generic preference-based measures, such as the EQ-5D instrument, for use in oral health (Rogers et al. 2022). As such, preference-based measures are being developed for specific conditions and age groups, either through adaptation of existing non-preference-based measures or created de novo (Goodwin and Green 2016).

To date, 2 preference-based measures have been developed specifically for oral health: the Caries Impacts and Experiences Questionnaire for Children (Rogers et al. 2022) and Early Childhood Oral Health Impact Scale 4 Dimensions (Hettiarachchi et al. 2022). Both were designed for a pediatric population, with the former being condition specific to dental caries and the latter for use in oral health more broadly. Such measures offer the potential to measure oral health–related QoL to generate QALYs. However, there remains a notable lack of available instruments, particularly for conditions such as periodontal disease and oral cancer.

## Other Approaches to Inform Oral Health Policy

Patient-reported outcome measures (PROMs) and patient-reported experience measures (PREMs) are increasingly used by health service providers for quality benchmarking and monitoring health outcomes for both individual and population groups. They have emerged since the mid-1900s and have remained important for health system performance measurement in England, the United States, and Australia (Bull and Callander 2022). PROMs and PREMs are the outcomes and experiences that matter to consumers and therefore should be given greater attention in oral health research and evaluation.

International benchmarking on dental caries and periodontal disease outcomes is made possible through the development of the Adult Oral Health Standard Set (AOHSS) developed by the International Consortium for Health Outcomes Measurement (Riordain et al. 2021). The AOHSS includes 25 items covering demographics, impact of oral health and oral function, a record of pain and oral hygiene practices, and financial implications of care. However, these PROMs and PREMs are not preference based, and further work is needed to consider their use in economic evaluations and policy decision-making.

A final alternative is the use of monetary valuations of health, such as willingness to pay (WTP) derived through contingent valuation (Tan et al. 2017) or discrete-choice experiments (Barber et al. 2018). The WTP threshold is a value the payer is prepared to spend for the cost and/or health benefit. While these measures allow comparison across (and beyond health), there are concerns about attaching monetary values to health and methodological issues with getting truly preference-based monetary values (Saadatfar and Jadidfard 2020).

## Implications for Oral Health Research and Policy

Information asymmetry between dental practitioners and consumers on dental treatment decisions can also affect outcomes and should be considered in policy decision-making. For example, promoting shared decision-making through dental professional competency standards and policies can help to bridge this gap. It facilitates considerations for patients’ “values, goals and preferences with the best available evidence about benefits, risks and uncertainties of disease management options, in order to reach the most appropriate healthcare decisions for that person” (Australian Commission on Safety and Quality in Health Care 2023).

Oral health research should consider outcome measures other than that conventionally measured by DMFT, to capture the health benefit beyond the dental caries experience. A broader scope of oral health impact on well-being can be assessed using burden of disease, preference-based QoL instruments, PROMs or PREMs, and monetary valuation.

## Author Contributions

T.M. Nguyen, H. Rogers, G.D. Taylor, U. Tonmukayakul, C. Vernazza, contributed to conception, design, drafted and critically revised the manuscript; C. Lin, M. Hall, H. Calache, contributed to data interpretation, critically revised the manuscript; All authors gave final approval and agree to be accountable for all aspects of the work.
